# Supplementary material for: Changes in the Spatial Structure of Synchronization Connections in EEG During Nocturnal Sleep Apnea
Source: Clocks Sleep. 2024 Dec 31;7(1):1. doi: 10.3390/clockssleep7010001 (PMC11755653; doi:10.3390/clockssleep7010001)
Supplement: Supplementary file 1 [file clockssleep-07-00001-s001.zip › clockssleep-3297074-supplementary.pdf]

Average values of WB synchronization in band $D f_1$, [0.25; 1] Hz, between pairs of EEG channels calculated for a group of healthy volunteers (value in top cell) and OSA patients (value in bottom cell)

	O1	O2	P4	P3	C4	C3	F4	F3	Fp1	Fp2	T6	T5	T4	T3	F8	F7	Pz	Cz	Fz	
O2	1.00	0.62	0.74	0.57	0.58	0.48	0.52	0.45	0.50	0.46	0.71	0.54	0.57	0.48	0.53	0.48	0.56	0.59	0.44	O2
	1.00	0.61	0.74	0.55	0.64	0.51	0.60	0.50	0.58	0.50	0.71	0.56	0.64	0.52	0.61	0.52	0.55	0.62	0.48	
O1	0.62	1.00	0.56	0.76	0.48	0.60	0.45	0.53	0.45	0.49	0.53	0.70	0.48	0.56	0.47	0.51	0.74	0.47	0.53	O1
	0.61	1.00	0.56	0.77	0.52	0.67	0.50	0.62	0.50	0.59	0.56	0.75	0.52	0.66	0.52	0.61	0.74	0.50	0.60	
P4	0.74	0.56	1.00	0.69	0.81	0.66	0.69	0.59	0.59	0.53	0.78	0.60	0.72	0.58	0.59	0.52	0.68	0.79	0.58	P4
	0.74	0.56	1.00	0.64	0.85	0.63	0.74	0.58	0.64	0.53	0.76	0.58	0.78	0.59	0.63	0.53	0.64	0.79	0.56	
P3	0.57	0.76	0.69	1.00	0.65	0.83	0.57	0.70	0.51	0.57	0.60	0.76	0.58	0.70	0.51	0.57	0.87	0.65	0.70	P3
	0.55	0.77	0.64	1.00	0.62	0.84	0.57	0.75	0.52	0.63	0.58	0.75	0.59	0.76	0.53	0.62	0.85	0.59	0.73	
C4	0.58	0.48	0.81	0.65	1.00	0.74	0.85	0.71	0.71	0.61	0.72	0.57	0.79	0.63	0.69	0.58	0.62	0.87	0.71	C4
	0.64	0.52	0.85	0.62	1.00	0.67	0.85	0.66	0.72	0.58	0.75	0.57	0.82	0.62	0.68	0.56	0.62	0.83	0.64	
C3	0.48	0.60	0.66	0.83	0.74	1.00	0.69	0.86	0.59	0.68	0.58	0.70	0.64	0.76	0.57	0.65	0.77	0.75	0.85	C3
	0.51	0.67	0.63	0.84	0.67	1.00	0.64	0.85	0.57	0.70	0.57	0.73	0.62	0.81	0.55	0.67	0.80	0.64	0.83	
F4	0.52	0.45	0.69	0.57	0.85	0.69	1.00	0.73	0.80	0.67	0.66	0.53	0.78	0.62	0.76	0.61	0.54	0.78	0.74	F4
	0.60	0.50	0.74	0.57	0.85	0.64	1.00	0.68	0.82	0.65	0.72	0.56	0.82	0.61	0.74	0.58	0.58	0.75	0.67	
F3	0.45	0.53	0.59	0.70	0.71	0.86	0.73	1.00	0.67	0.81	0.54	0.64	0.63	0.77	0.60	0.73	0.64	0.70	0.88	F3
	0.50	0.62	0.58	0.75	0.66	0.85	0.68	1.00	0.64	0.82	0.58	0.71	0.64	0.82	0.59	0.75	0.72	0.61	0.85	
Fp2	0.50	0.45	0.59	0.51	0.71	0.59	0.80	0.67	1.00	0.74	0.60	0.51	0.74	0.59	0.78	0.62	0.49	0.64	0.68	Fp2
	0.58	0.50	0.64	0.52	0.72	0.57	0.82	0.64	1.00	0.70	0.68	0.54	0.76	0.57	0.77	0.57	0.53	0.64	0.62	
Fp1	0.46	0.49	0.53	0.57	0.61	0.68	0.67	0.81	0.74	1.00	0.52	0.60	0.60	0.72	0.62	0.78	0.53	0.59	0.75	Fp1
	0.50	0.59	0.53	0.63	0.58	0.70	0.65	0.82	0.70	1.00	0.55	0.66	0.59	0.75	0.60	0.77	0.62	0.55	0.74	
T6	0.71	0.53	0.78	0.60	0.72	0.58	0.66	0.54	0.60	0.52	1.00	0.60	0.77	0.58	0.66	0.52	0.58	0.66	0.54	T6
	0.71	0.56	0.76	0.58	0.75	0.57	0.72	0.58	0.68	0.55	1.00	0.59	0.81	0.59	0.73	0.55	0.57	0.66	0.55	
T5	0.54	0.70	0.60	0.76	0.57	0.70	0.53	0.64	0.51	0.60	0.60	1.00	0.56	0.78	0.51	0.66	0.67	0.56	0.61	T5
	0.56	0.75	0.58	0.75	0.57	0.73	0.56	0.71	0.54	0.66	0.59	1.00	0.57	0.80	0.55	0.71	0.70	0.53	0.66	
T4	0.57	0.48	0.72	0.58	0.79	0.64	0.78	0.63	0.74	0.60	0.77	0.56	1.00	0.63	0.83	0.60	0.55	0.70	0.65	T4
	0.64	0.52	0.78	0.59	0.82	0.62	0.82	0.64	0.76	0.59	0.81	0.57	1.00	0.62	0.81	0.57	0.59	0.71	0.62	
T3	0.48	0.56	0.58	0.70	0.63	0.76	0.62	0.77	0.59	0.72	0.58	0.78	0.63	1.00	0.59	0.82	0.61	0.63	0.71	T3
	0.52	0.66	0.59	0.76	0.62	0.81	0.61	0.82	0.57	0.75	0.59	0.80	0.62	1.00	0.57	0.82	0.70	0.59	0.75	
F8	0.53	0.47	0.59	0.51	0.69	0.57	0.76	0.60	0.78	0.62	0.66	0.51	0.83	0.59	1.00	0.61	0.50	0.61	0.63	F8
	0.61	0.52	0.63	0.53	0.68	0.55	0.74	0.59	0.77	0.60	0.73	0.55	0.81	0.57	1.00	0.58	0.53	0.60	0.58	
F7	0.48	0.51	0.52	0.57	0.58	0.65	0.61	0.73	0.62	0.78	0.52	0.66	0.60	0.82	0.61	1.00	0.52	0.58	0.66	F7
	0.52	0.61	0.53	0.62	0.56	0.67	0.58	0.75	0.57	0.77	0.55	0.71	0.57	0.82	0.58	1.00	0.60	0.53	0.67	
Pz	0.56	0.74	0.68	0.87	0.62	0.77	0.54	0.64	0.49	0.53	0.58	0.67	0.55	0.61	0.50	0.52	1.00	0.62	0.68	Pz
	0.55	0.74	0.64	0.85	0.62	0.80	0.58	0.72	0.53	0.62	0.57	0.70	0.59	0.70	0.53	0.60	1.00	0.59	0.73	
Cz	0.59	0.47	0.79	0.65	0.87	0.75	0.78	0.70	0.64	0.59	0.66	0.56	0.70	0.63	0.61	0.58	0.62	1.00	0.69	Cz
	0.62	0.50	0.79	0.59	0.83	0.64	0.75	0.61	0.64	0.55	0.66	0.53	0.71	0.59	0.60	0.53	0.59	1.00	0.60	
Fz	0.44	0.53	0.58	0.70	0.71	0.85	0.74	0.88	0.68	0.75	0.54	0.61	0.65	0.71	0.63	0.66	0.68	0.69	1.00	Fz
	0.48	0.60	0.56	0.73	0.64	0.83	0.67	0.85	0.62	0.74	0.55	0.66	0.62	0.75	0.58	0.67	0.73	0.60	1.00	
	O1	O2	P4	P3	C4	C3	F4	F3	Fp1	Fp2	T6	T5	T4	T3	F8	F7	Pz	Cz	Fz	

Average values of WB synchronization in band $D\ f_2$, [1; 4] Hz, between pairs of EEG channels calculated for a group of healthy volunteers (value in top cell) and OSA patients (value in bottom cell)

	O1	O2	P4	P3	C4	C3	F4	F3	Fp1	Fp2	T6	T5	T4	T3	F8	F7	Pz	Cz	Fz	
O2	1.00	0.61	0.73	0.54	0.49	0.39	0.39	0.33	0.35	0.31	0.67	0.47	0.46	0.36	0.36	0.31	0.56	0.49	0.32	O2
	1.00	0.53	0.72	0.44	0.55	0.37	0.45	0.34	0.39	0.34	0.67	0.42	0.51	0.36	0.40	0.35	0.46	0.51	0.33	
O1	0.61	1.00	0.55	0.77	0.40	0.55	0.33	0.43	0.31	0.36	0.48	0.69	0.36	0.50	0.32	0.37	0.74	0.41	0.43	O1
	0.53	1.00	0.46	0.75	0.38	0.59	0.33	0.49	0.32	0.42	0.42	0.70	0.36	0.55	0.33	0.43	0.72	0.36	0.46	
P4	0.73	0.55	1.00	0.73	0.82	0.68	0.67	0.58	0.54	0.46	0.80	0.59	0.74	0.56	0.55	0.44	0.74	0.79	0.57	P4
	0.72	0.46	1.00	0.62	0.86	0.59	0.72	0.51	0.58	0.41	0.79	0.49	0.79	0.51	0.55	0.38	0.63	0.79	0.51	
P3	0.54	0.77	0.73	1.00	0.66	0.84	0.54	0.70	0.43	0.53	0.59	0.79	0.55	0.74	0.43	0.55	0.89	0.68	0.68	P3
	0.44	0.75	0.62	1.00	0.59	0.87	0.50	0.73	0.41	0.58	0.50	0.77	0.52	0.79	0.39	0.56	0.88	0.57	0.71	
C4	0.49	0.40	0.82	0.66	1.00	0.79	0.87	0.75	0.74	0.62	0.70	0.54	0.83	0.64	0.72	0.57	0.64	0.91	0.76	C4
	0.55	0.38	0.86	0.59	1.00	0.68	0.88	0.64	0.75	0.54	0.74	0.48	0.87	0.58	0.68	0.46	0.60	0.87	0.65	
C3	0.39	0.55	0.68	0.84	0.79	1.00	0.71	0.90	0.60	0.73	0.56	0.70	0.65	0.81	0.57	0.70	0.78	0.80	0.87	C3
	0.37	0.59	0.59	0.87	0.68	1.00	0.63	0.89	0.54	0.74	0.50	0.73	0.60	0.86	0.49	0.68	0.83	0.66	0.85	
F4	0.39	0.33	0.67	0.54	0.87	0.71	1.00	0.77	0.86	0.71	0.60	0.46	0.79	0.61	0.78	0.60	0.51	0.80	0.78	F4
	0.45	0.33	0.72	0.50	0.88	0.63	1.00	0.69	0.87	0.65	0.67	0.45	0.84	0.57	0.76	0.52	0.51	0.79	0.70	
F3	0.33	0.43	0.58	0.70	0.75	0.90	0.77	1.00	0.73	0.89	0.49	0.60	0.65	0.80	0.63	0.78	0.63	0.75	0.93	F3
	0.34	0.49	0.51	0.73	0.64	0.89	0.69	1.00	0.65	0.88	0.48	0.67	0.60	0.85	0.55	0.77	0.71	0.61	0.90	
Fp2	0.35	0.31	0.54	0.43	0.74	0.60	0.86	0.73	1.00	0.79	0.50	0.39	0.73	0.56	0.81	0.62	0.40	0.67	0.73	Fp2
	0.39	0.32	0.58	0.41	0.75	0.54	0.87	0.65	1.00	0.71	0.60	0.41	0.77	0.52	0.79	0.54	0.42	0.65	0.65	
Fp1	0.31	0.36	0.46	0.53	0.62	0.73	0.71	0.89	0.79	1.00	0.41	0.49	0.58	0.71	0.63	0.79	0.49	0.59	0.83	Fp1
	0.34	0.42	0.41	0.58	0.54	0.74	0.65	0.88	0.71	1.00	0.43	0.58	0.55	0.76	0.57	0.80	0.58	0.50	0.82	
T6	0.67	0.48	0.80	0.59	0.70	0.56	0.60	0.49	0.50	0.41	1.00	0.55	0.77	0.51	0.60	0.41	0.60	0.62	0.50	T6
	0.67	0.42	0.79	0.50	0.74	0.50	0.67	0.48	0.60	0.43	1.00	0.47	0.81	0.47	0.66	0.40	0.51	0.63	0.47	
T5	0.47	0.69	0.59	0.79	0.54	0.70	0.46	0.60	0.39	0.49	0.55	1.00	0.50	0.79	0.40	0.61	0.67	0.56	0.56	T5
	0.42	0.70	0.49	0.77	0.48	0.73	0.45	0.67	0.41	0.58	0.47	1.00	0.46	0.81	0.39	0.66	0.69	0.47	0.60	
T4	0.46	0.36	0.74	0.55	0.83	0.65	0.79	0.65	0.73	0.58	0.77	0.50	1.00	0.61	0.84	0.56	0.55	0.72	0.67	T4
	0.51	0.36	0.79	0.52	0.87	0.60	0.84	0.60	0.77	0.55	0.81	0.46	1.00	0.56	0.82	0.47	0.53	0.74	0.61	
T3	0.36	0.50	0.56	0.74	0.64	0.81	0.61	0.80	0.56	0.71	0.51	0.79	0.61	1.00	0.55	0.84	0.62	0.65	0.73	T3
	0.36	0.55	0.51	0.79	0.58	0.86	0.57	0.85	0.52	0.76	0.47	0.81	0.56	1.00	0.48	0.83	0.70	0.56	0.76	
F8	0.36	0.32	0.55	0.43	0.72	0.57	0.78	0.63	0.81	0.63	0.60	0.40	0.84	0.55	1.00	0.58	0.42	0.61	0.65	F8
	0.40	0.33	0.55	0.39	0.68	0.49	0.76	0.55	0.79	0.57	0.66	0.39	0.82	0.48	1.00	0.50	0.40	0.57	0.56	
F7	0.31	0.37	0.44	0.55	0.57	0.70	0.60	0.78	0.62	0.79	0.41	0.61	0.56	0.84	0.58	1.00	0.46	0.57	0.70	F7
	0.35	0.43	0.38	0.56	0.46	0.68	0.52	0.77	0.54	0.80	0.40	0.66	0.47	0.83	0.50	1.00	0.51	0.44	0.68	
Pz	0.56	0.74	0.74	0.89	0.64	0.78	0.51	0.63	0.40	0.49	0.60	0.67	0.55	0.62	0.42	0.46	1.00	0.64	0.65	Pz
	0.46	0.72	0.63	0.88	0.60	0.83	0.51	0.71	0.42	0.58	0.51	0.69	0.53	0.70	0.40	0.51	1.00	0.57	0.71	
Cz	0.49	0.41	0.79	0.68	0.91	0.80	0.80	0.75	0.67	0.59	0.62	0.56	0.72	0.65	0.61	0.57	0.64	1.00	0.74	Cz
	0.51	0.36	0.79	0.57	0.87	0.66	0.79	0.61	0.65	0.50	0.63	0.47	0.74	0.56	0.57	0.44	0.57	1.00	0.61	
Fz	0.32	0.43	0.57	0.68	0.76	0.87	0.78	0.93	0.73	0.83	0.50	0.56	0.67	0.73	0.65	0.70	0.65	0.74	1.00	Fz
	0.33	0.46	0.51	0.71	0.65	0.85	0.70	0.90	0.65	0.82	0.47	0.60	0.61	0.76	0.56	0.68	0.71	0.61	1.00	
	O1	O2	P4	P3	C4	C3	F4	F3	Fp1	Fp2	T6	T5	T4	T3	F8	F7	Pz	Cz	Fz	

Average values of WB synchronization in band $D\,f_3$, [4; 8] Hz, between pairs of EEG channels calculated for a group of healthy volunteers (value in top cell) and OSA patients (value in bottom cell)

	O1	O2	P4	P3	C4	C3	F4	F3	Fp1	Fp2	T6	T5	T4	T3	F8	F7	Pz	Cz	Fz	
O2	1.00	0.29	0.37	0.25	0.24	0.19	0.19	0.17	0.18	0.17	0.34	0.21	0.23	0.17	0.18	0.17	0.27	0.23	0.17	O2
	1.00	0.26	0.37	0.21	0.27	0.19	0.22	0.19	0.19	0.21	0.34	0.21	0.25	0.19	0.20	0.22	0.22	0.25	0.19	
O1	0.29	1.00	0.27	0.38	0.19	0.26	0.16	0.21	0.16	0.18	0.22	0.34	0.17	0.24	0.16	0.19	0.36	0.20	0.20	O1
	0.26	1.00	0.23	0.39	0.19	0.29	0.17	0.24	0.17	0.22	0.21	0.35	0.18	0.27	0.18	0.22	0.36	0.18	0.23	
P4	0.37	0.27	1.00	0.34	0.41	0.31	0.32	0.26	0.26	0.21	0.40	0.26	0.37	0.25	0.27	0.20	0.36	0.38	0.26	P4
	0.37	0.23	1.00	0.29	0.43	0.27	0.35	0.23	0.28	0.20	0.40	0.23	0.40	0.23	0.27	0.19	0.30	0.39	0.23	
P3	0.25	0.38	0.34	1.00	0.31	0.42	0.25	0.34	0.20	0.27	0.26	0.39	0.25	0.37	0.20	0.28	0.44	0.32	0.32	P3
	0.21	0.39	0.29	1.00	0.27	0.44	0.23	0.36	0.20	0.30	0.23	0.40	0.24	0.40	0.19	0.29	0.45	0.27	0.35	
C4	0.24	0.19	0.41	0.31	1.00	0.37	0.44	0.35	0.38	0.29	0.34	0.24	0.42	0.29	0.37	0.26	0.30	0.45	0.36	C4
	0.27	0.19	0.43	0.27	1.00	0.31	0.44	0.29	0.38	0.25	0.37	0.22	0.44	0.26	0.35	0.21	0.28	0.44	0.29	
C3	0.19	0.26	0.31	0.42	0.37	1.00	0.34	0.45	0.29	0.38	0.25	0.34	0.31	0.41	0.27	0.36	0.39	0.38	0.43	C3
	0.19	0.29	0.27	0.44	0.31	1.00	0.29	0.45	0.26	0.39	0.23	0.37	0.27	0.44	0.24	0.36	0.42	0.31	0.43	
F4	0.19	0.16	0.32	0.25	0.44	0.34	1.00	0.36	0.44	0.34	0.28	0.21	0.39	0.28	0.39	0.28	0.23	0.40	0.38	F4
	0.22	0.17	0.35	0.23	0.44	0.29	1.00	0.31	0.45	0.29	0.31	0.21	0.42	0.25	0.39	0.24	0.23	0.40	0.32	
F3	0.17	0.21	0.26	0.34	0.35	0.45	0.36	1.00	0.35	0.46	0.22	0.28	0.30	0.39	0.30	0.40	0.31	0.35	0.47	F3
	0.19	0.24	0.23	0.36	0.29	0.45	0.31	1.00	0.30	0.46	0.22	0.32	0.27	0.42	0.26	0.40	0.36	0.28	0.46	
Fp2	0.18	0.16	0.26	0.20	0.38	0.29	0.44	0.35	1.00	0.37	0.22	0.18	0.35	0.26	0.40	0.29	0.19	0.35	0.35	Fp2
	0.19	0.17	0.28	0.20	0.38	0.26	0.45	0.30	1.00	0.32	0.27	0.19	0.38	0.24	0.40	0.25	0.20	0.35	0.30	
Fp1	0.17	0.18	0.21	0.27	0.29	0.38	0.34	0.46	0.37	1.00	0.19	0.23	0.27	0.35	0.30	0.40	0.25	0.28	0.43	Fp1
	0.21	0.22	0.20	0.30	0.25	0.39	0.29	0.46	0.32	1.00	0.20	0.29	0.24	0.38	0.27	0.41	0.31	0.23	0.43	
T6	0.34	0.22	0.40	0.26	0.34	0.25	0.28	0.22	0.22	0.19	1.00	0.23	0.38	0.22	0.28	0.18	0.28	0.29	0.23	T6
	0.34	0.21	0.40	0.23	0.37	0.23	0.31	0.22	0.27	0.20	1.00	0.22	0.41	0.22	0.31	0.20	0.25	0.30	0.21	
T5	0.21	0.34	0.26	0.39	0.24	0.34	0.21	0.28	0.18	0.23	0.23	1.00	0.22	0.40	0.18	0.30	0.31	0.26	0.26	T5
	0.21	0.35	0.23	0.40	0.22	0.37	0.21	0.32	0.19	0.29	0.22	1.00	0.21	0.41	0.19	0.32	0.34	0.22	0.29	
T4	0.23	0.17	0.37	0.25	0.42	0.31	0.39	0.30	0.35	0.27	0.38	0.22	1.00	0.27	0.42	0.25	0.26	0.35	0.31	T4
	0.25	0.18	0.40	0.24	0.44	0.27	0.42	0.27	0.38	0.24	0.41	0.21	1.00	0.24	0.42	0.21	0.25	0.37	0.28	
T3	0.17	0.24	0.25	0.37	0.29	0.41	0.28	0.39	0.26	0.35	0.22	0.40	0.27	1.00	0.25	0.42	0.30	0.31	0.35	T3
	0.19	0.27	0.23	0.40	0.26	0.44	0.25	0.42	0.24	0.38	0.22	0.41	0.24	1.00	0.22	0.43	0.35	0.26	0.37	
F8	0.18	0.16	0.27	0.20	0.37	0.27	0.39	0.30	0.40	0.30	0.28	0.18	0.42	0.25	1.00	0.26	0.20	0.31	0.31	F8
	0.20	0.18	0.27	0.19	0.35	0.24	0.39	0.26	0.40	0.27	0.31	0.19	0.42	0.22	1.00	0.23	0.20	0.29	0.27	
F7	0.17	0.19	0.20	0.28	0.26	0.36	0.28	0.40	0.29	0.40	0.18	0.30	0.25	0.42	0.26	1.00	0.24	0.27	0.35	F7
	0.22	0.22	0.19	0.29	0.21	0.36	0.24	0.40	0.25	0.41	0.20	0.32	0.21	0.43	0.23	1.00	0.27	0.21	0.35	
Pz	0.27	0.36	0.36	0.44	0.30	0.39	0.23	0.31	0.19	0.25	0.28	0.31	0.26	0.30	0.20	0.24	1.00	0.30	0.32	Pz
	0.22	0.36	0.30	0.45	0.28	0.42	0.23	0.36	0.20	0.31	0.25	0.34	0.25	0.35	0.20	0.27	1.00	0.26	0.36	
Cz	0.23	0.20	0.38	0.32	0.45	0.38	0.40	0.35	0.35	0.28	0.29	0.26	0.35	0.31	0.31	0.27	0.30	1.00	0.35	Cz
	0.25	0.18	0.39	0.27	0.44	0.31	0.40	0.28	0.35	0.23	0.30	0.22	0.37	0.26	0.29	0.21	0.26	1.00	0.28	
Fz	0.17	0.20	0.26	0.32	0.36	0.43	0.38	0.47	0.35	0.43	0.23	0.26	0.31	0.35	0.31	0.35	0.32	0.35	1.00	Fz
	0.19	0.23	0.23	0.35	0.29	0.43	0.32	0.46	0.30	0.43	0.21	0.29	0.28	0.37	0.27	0.35	0.36	0.28	1.00	
	O1	O2	P4	P3	C4	C3	F4	F3	Fp1	Fp2	T6	T5	T4	T3	F8	F7	Pz	Cz	Fz	

Average values of WB synchronization in band $D\ f_4$, [8; 12] Hz, between pairs of EEG channels calculated for a group of healthy volunteers (value in top cell) and OSA patients (value in bottom cell)

	O1	O2	P4	P3	C4	C3	F4	F3	Fp1	Fp2	T6	T5	T4	T3	F8	F7	Pz	Cz	Fz	
O2	1.00	0.29	0.37	0.26	0.26	0.21	0.22	0.20	0.21	0.21	0.35	0.23	0.25	0.20	0.22	0.21	0.27	0.25	0.20	O2
	1.00	0.27	0.38	0.24	0.30	0.23	0.26	0.24	0.24	0.25	0.36	0.24	0.28	0.23	0.24	0.26	0.25	0.28	0.22	
O1	0.29	1.00	0.27	0.39	0.21	0.28	0.19	0.23	0.20	0.22	0.23	0.35	0.20	0.27	0.20	0.23	0.36	0.22	0.23	O1
	0.27	1.00	0.25	0.40	0.22	0.32	0.21	0.28	0.22	0.26	0.23	0.38	0.22	0.30	0.22	0.27	0.38	0.21	0.26	
P4	0.37	0.27	1.00	0.33	0.41	0.30	0.32	0.26	0.26	0.22	0.40	0.26	0.37	0.25	0.29	0.22	0.36	0.37	0.26	P4
	0.38	0.25	1.00	0.28	0.43	0.27	0.35	0.24	0.29	0.22	0.41	0.24	0.40	0.24	0.29	0.22	0.29	0.39	0.23	
P3	0.26	0.39	0.33	1.00	0.30	0.42	0.25	0.33	0.22	0.28	0.26	0.39	0.25	0.37	0.22	0.29	0.44	0.31	0.32	P3
	0.24	0.40	0.28	1.00	0.26	0.44	0.23	0.37	0.22	0.32	0.24	0.41	0.25	0.41	0.22	0.31	0.45	0.26	0.35	
C4	0.26	0.21	0.41	0.30	1.00	0.35	0.44	0.33	0.38	0.28	0.34	0.24	0.42	0.28	0.37	0.26	0.30	0.45	0.34	C4
	0.30	0.22	0.43	0.26	1.00	0.29	0.44	0.27	0.39	0.25	0.37	0.23	0.45	0.25	0.37	0.23	0.27	0.44	0.28	
C3	0.21	0.28	0.30	0.42	0.35	1.00	0.32	0.45	0.28	0.37	0.25	0.34	0.29	0.41	0.26	0.37	0.39	0.37	0.42	C3
	0.23	0.32	0.27	0.44	0.29	1.00	0.27	0.45	0.25	0.40	0.24	0.37	0.26	0.44	0.24	0.37	0.42	0.29	0.43	
F4	0.22	0.19	0.32	0.25	0.44	0.32	1.00	0.35	0.44	0.32	0.28	0.22	0.38	0.27	0.39	0.28	0.24	0.40	0.36	F4
	0.26	0.21	0.35	0.23	0.44	0.27	1.00	0.29	0.46	0.27	0.32	0.22	0.41	0.24	0.40	0.24	0.24	0.41	0.29	
F3	0.20	0.23	0.26	0.33	0.33	0.45	0.35	1.00	0.33	0.46	0.22	0.28	0.28	0.38	0.29	0.40	0.31	0.34	0.47	F3
	0.24	0.28	0.24	0.37	0.27	0.45	0.29	1.00	0.28	0.47	0.23	0.33	0.26	0.42	0.26	0.41	0.37	0.27	0.46	
Fp2	0.21	0.20	0.26	0.22	0.38	0.28	0.44	0.33	1.00	0.35	0.24	0.20	0.34	0.25	0.40	0.28	0.21	0.35	0.34	Fp2
	0.24	0.22	0.29	0.22	0.39	0.25	0.46	0.28	1.00	0.29	0.28	0.22	0.37	0.23	0.41	0.25	0.22	0.36	0.28	
Fp1	0.21	0.22	0.22	0.28	0.28	0.37	0.32	0.46	0.35	1.00	0.21	0.24	0.26	0.34	0.29	0.40	0.26	0.28	0.43	Fp1
	0.25	0.26	0.22	0.32	0.25	0.40	0.27	0.47	0.29	1.00	0.23	0.30	0.24	0.38	0.26	0.42	0.33	0.24	0.44	
T6	0.35	0.23	0.40	0.26	0.34	0.25	0.28	0.22	0.24	0.21	1.00	0.24	0.38	0.22	0.30	0.20	0.28	0.29	0.23	T6
	0.36	0.23	0.41	0.24	0.37	0.24	0.32	0.23	0.28	0.23	1.00	0.24	0.41	0.23	0.32	0.23	0.25	0.31	0.23	
T5	0.23	0.35	0.26	0.39	0.24	0.34	0.22	0.28	0.20	0.24	0.24	1.00	0.22	0.40	0.20	0.31	0.31	0.26	0.26	T5
	0.24	0.38	0.24	0.41	0.23	0.37	0.22	0.33	0.22	0.30	0.24	1.00	0.22	0.41	0.22	0.34	0.35	0.23	0.29	
T4	0.25	0.20	0.37	0.25	0.42	0.29	0.38	0.28	0.34	0.26	0.38	0.22	1.00	0.26	0.42	0.25	0.26	0.35	0.30	T4
	0.28	0.22	0.40	0.25	0.45	0.26	0.41	0.26	0.37	0.24	0.41	0.22	1.00	0.24	0.42	0.23	0.25	0.37	0.26	
T3	0.20	0.27	0.25	0.37	0.28	0.41	0.27	0.38	0.25	0.34	0.22	0.40	0.26	1.00	0.24	0.42	0.30	0.30	0.34	T3
	0.23	0.30	0.24	0.41	0.25	0.44	0.24	0.42	0.23	0.38	0.23	0.41	0.24	1.00	0.23	0.43	0.36	0.25	0.37	
F8	0.22	0.20	0.29	0.22	0.37	0.26	0.39	0.29	0.40	0.29	0.30	0.20	0.42	0.24	1.00	0.26	0.22	0.31	0.31	F8
	0.24	0.22	0.29	0.22	0.37	0.24	0.40	0.26	0.41	0.26	0.32	0.22	0.42	0.23	1.00	0.24	0.22	0.31	0.26	
F7	0.21	0.23	0.22	0.29	0.26	0.37	0.28	0.40	0.28	0.40	0.20	0.31	0.25	0.42	0.26	1.00	0.25	0.27	0.35	F7
	0.26	0.27	0.22	0.31	0.23	0.37	0.24	0.41	0.25	0.42	0.23	0.34	0.23	0.43	0.24	1.00	0.30	0.22	0.36	
Pz	0.27	0.36	0.36	0.44	0.30	0.39	0.24	0.31	0.21	0.26	0.28	0.31	0.26	0.30	0.22	0.25	1.00	0.29	0.32	Pz
	0.25	0.38	0.29	0.45	0.27	0.42	0.24	0.37	0.22	0.33	0.25	0.35	0.25	0.36	0.22	0.30	1.00	0.26	0.36	
Cz	0.25	0.22	0.37	0.31	0.45	0.37	0.40	0.34	0.35	0.28	0.29	0.26	0.35	0.30	0.31	0.27	0.29	1.00	0.34	Cz
	0.28	0.21	0.39	0.26	0.44	0.29	0.41	0.27	0.36	0.24	0.31	0.23	0.37	0.25	0.31	0.22	0.26	1.00	0.27	
Fz	0.20	0.23	0.26	0.32	0.34	0.42	0.36	0.47	0.34	0.43	0.23	0.26	0.30	0.34	0.31	0.35	0.32	0.34	1.00	Fz
	0.22	0.26	0.23	0.35	0.28	0.43	0.29	0.46	0.28	0.44	0.23	0.29	0.26	0.37	0.26	0.36	0.36	0.27	1.00	
	O1	O2	P4	P3	C4	C3	F4	F3	Fp1	Fp2	T6	T5	T4	T3	F8	F7	Pz	Cz	Fz	

Average values of WB synchronization in band $D\,f_5$, [12; 20] Hz, between pairs of EEG channels calculated for a group of healthy volunteers (value in top cell) and OSA patients (value in bottom cell)

	O1	O2	P4	P3	C4	C3	F4	F3	Fp1	Fp2	T6	T5	T4	T3	F8	F7	Pz	Cz	Fz	
O2	1.00	0.26	0.38	0.24	0.27	0.21	0.23	0.20	0.21	0.21	0.35	0.21	0.27	0.20	0.22	0.20	0.25	0.26	0.20	O2
	1.00	0.25	0.39	0.23	0.31	0.23	0.27	0.24	0.25	0.26	0.37	0.24	0.30	0.24	0.26	0.26	0.24	0.30	0.23	
O1	0.26	1.00	0.24	0.39	0.20	0.30	0.19	0.25	0.19	0.23	0.21	0.36	0.19	0.29	0.19	0.24	0.36	0.20	0.24	O1
	0.25	1.00	0.23	0.41	0.21	0.34	0.21	0.30	0.22	0.29	0.22	0.39	0.21	0.33	0.22	0.29	0.39	0.21	0.29	
P4	0.38	0.24	1.00	0.28	0.41	0.26	0.32	0.23	0.27	0.21	0.39	0.22	0.37	0.22	0.29	0.20	0.32	0.38	0.23	P4
	0.39	0.23	1.00	0.25	0.44	0.24	0.36	0.23	0.30	0.22	0.41	0.23	0.41	0.23	0.31	0.22	0.26	0.40	0.22	
P3	0.24	0.39	0.28	1.00	0.26	0.42	0.22	0.34	0.20	0.29	0.22	0.39	0.22	0.37	0.20	0.30	0.44	0.28	0.33	P3
	0.23	0.41	0.25	1.00	0.24	0.45	0.22	0.38	0.21	0.34	0.22	0.41	0.22	0.42	0.21	0.34	0.45	0.24	0.37	
C4	0.27	0.20	0.41	0.26	1.00	0.30	0.43	0.28	0.36	0.24	0.33	0.21	0.40	0.23	0.35	0.22	0.27	0.44	0.30	C4
	0.31	0.21	0.44	0.24	1.00	0.25	0.44	0.24	0.38	0.23	0.37	0.22	0.44	0.23	0.36	0.22	0.24	0.43	0.25	
C3	0.21	0.30	0.26	0.42	0.30	1.00	0.27	0.45	0.24	0.37	0.22	0.34	0.24	0.40	0.22	0.36	0.39	0.33	0.42	C3
	0.23	0.34	0.24	0.45	0.25	1.00	0.24	0.46	0.23	0.40	0.22	0.38	0.23	0.44	0.22	0.38	0.43	0.26	0.43	
F4	0.23	0.19	0.32	0.22	0.43	0.27	1.00	0.29	0.43	0.27	0.27	0.19	0.37	0.23	0.37	0.23	0.22	0.39	0.32	F4
	0.27	0.21	0.36	0.22	0.44	0.24	1.00	0.25	0.44	0.24	0.32	0.21	0.41	0.22	0.39	0.22	0.22	0.40	0.26	
F3	0.20	0.25	0.23	0.34	0.28	0.45	0.29	1.00	0.28	0.45	0.20	0.28	0.24	0.37	0.24	0.39	0.32	0.30	0.46	F3
	0.24	0.30	0.23	0.38	0.24	0.46	0.25	1.00	0.25	0.46	0.23	0.34	0.23	0.42	0.23	0.41	0.38	0.24	0.46	
Fp2	0.21	0.19	0.27	0.20	0.36	0.24	0.43	0.28	1.00	0.29	0.24	0.18	0.33	0.22	0.38	0.24	0.19	0.34	0.30	Fp2
	0.25	0.22	0.30	0.21	0.38	0.23	0.44	0.25	1.00	0.25	0.29	0.21	0.37	0.22	0.40	0.23	0.21	0.35	0.25	
Fp1	0.21	0.23	0.21	0.29	0.24	0.37	0.27	0.45	0.29	1.00	0.19	0.25	0.22	0.33	0.24	0.39	0.28	0.24	0.42	Fp1
	0.26	0.29	0.22	0.34	0.23	0.40	0.24	0.46	0.25	1.00	0.23	0.32	0.23	0.39	0.24	0.42	0.35	0.22	0.44	
T6	0.35	0.21	0.39	0.22	0.33	0.22	0.27	0.20	0.24	0.19	1.00	0.21	0.37	0.20	0.29	0.19	0.25	0.29	0.21	T6
	0.37	0.22	0.41	0.22	0.37	0.22	0.32	0.23	0.29	0.23	1.00	0.23	0.41	0.22	0.33	0.23	0.23	0.31	0.22	
T5	0.21	0.36	0.22	0.39	0.21	0.34	0.19	0.28	0.18	0.25	0.21	1.00	0.19	0.39	0.18	0.31	0.32	0.23	0.26	T5
	0.24	0.39	0.23	0.41	0.22	0.38	0.21	0.34	0.21	0.32	0.23	1.00	0.22	0.42	0.22	0.36	0.37	0.21	0.32	
T4	0.27	0.19	0.37	0.22	0.40	0.24	0.37	0.24	0.33	0.22	0.37	0.19	1.00	0.22	0.40	0.21	0.23	0.34	0.26	T4
	0.30	0.21	0.41	0.22	0.44	0.23	0.41	0.23	0.37	0.23	0.41	0.22	1.00	0.22	0.41	0.22	0.23	0.36	0.23	
T3	0.20	0.29	0.22	0.37	0.23	0.40	0.23	0.37	0.22	0.33	0.20	0.39	0.22	1.00	0.21	0.41	0.31	0.26	0.33	T3
	0.24	0.33	0.23	0.42	0.23	0.44	0.22	0.42	0.22	0.39	0.22	0.42	0.22	1.00	0.22	0.43	0.38	0.23	0.38	
F8	0.22	0.19	0.29	0.20	0.35	0.22	0.37	0.24	0.38	0.24	0.29	0.18	0.40	0.21	1.00	0.22	0.20	0.30	0.26	F8
	0.26	0.22	0.31	0.21	0.36	0.22	0.39	0.23	0.40	0.24	0.33	0.22	0.41	0.22	1.00	0.23	0.21	0.31	0.23	
F7	0.20	0.24	0.20	0.30	0.22	0.36	0.23	0.39	0.24	0.39	0.19	0.31	0.21	0.41	0.22	1.00	0.26	0.23	0.34	F7
	0.26	0.29	0.22	0.34	0.22	0.38	0.22	0.41	0.23	0.42	0.23	0.36	0.22	0.43	0.23	1.00	0.32	0.21	0.37	
Pz	0.25	0.36	0.32	0.44	0.27	0.39	0.22	0.32	0.19	0.28	0.25	0.32	0.23	0.31	0.20	0.26	1.00	0.27	0.34	Pz
	0.24	0.39	0.26	0.45	0.24	0.43	0.22	0.38	0.21	0.35	0.23	0.37	0.23	0.38	0.21	0.32	1.00	0.24	0.38	
Cz	0.26	0.20	0.38	0.28	0.44	0.33	0.39	0.30	0.34	0.24	0.29	0.23	0.34	0.26	0.30	0.23	0.27	1.00	0.30	Cz
	0.30	0.21	0.40	0.24	0.43	0.26	0.40	0.24	0.35	0.22	0.31	0.21	0.36	0.23	0.31	0.21	0.24	1.00	0.24	
Fz	0.20	0.24	0.23	0.33	0.30	0.42	0.32	0.46	0.30	0.42	0.21	0.26	0.26	0.33	0.26	0.34	0.34	0.30	1.00	Fz
	0.23	0.29	0.22	0.37	0.25	0.43	0.26	0.46	0.25	0.44	0.22	0.32	0.23	0.38	0.23	0.37	0.38	0.24	1.00	
	O1	O2	P4	P3	C4	C3	F4	F3	Fp1	Fp2	T6	T5	T4	T3	F8	F7	Pz	Cz	Fz	

Average values of WB synchronization in band $D\ f_6$, [20; 30] Hz, between pairs of EEG channels calculated for a group of healthy volunteers (value in top cell) and OSA patients (value in bottom cell)

	O1	O2	P4	P3	C4	C3	F4	F3	Fp1	Fp2	T6	T5	T4	T3	F8	F7	Pz	Cz	Fz	
O2	1.00	0.22	0.38	0.21	0.29	0.20	0.24	0.20	0.23	0.20	0.35	0.19	0.28	0.19	0.24	0.20	0.23	0.28	0.19	O2
	1.00	0.23	0.39	0.23	0.32	0.22	0.28	0.23	0.26	0.23	0.37	0.22	0.31	0.22	0.26	0.23	0.23	0.30	0.22	
O1	0.22	1.00	0.21	0.39	0.19	0.31	0.18	0.26	0.19	0.24	0.19	0.36	0.19	0.30	0.18	0.26	0.36	0.19	0.26	O1
	0.23	1.00	0.22	0.41	0.21	0.34	0.20	0.31	0.21	0.29	0.21	0.39	0.21	0.34	0.21	0.30	0.39	0.20	0.30	
P4	0.38	0.21	1.00	0.24	0.41	0.23	0.31	0.21	0.27	0.20	0.38	0.20	0.37	0.20	0.29	0.19	0.27	0.37	0.21	P4
	0.39	0.22	1.00	0.24	0.44	0.23	0.35	0.22	0.30	0.22	0.40	0.22	0.40	0.22	0.31	0.22	0.25	0.39	0.22	
P3	0.21	0.39	0.24	1.00	0.23	0.42	0.20	0.34	0.19	0.29	0.20	0.38	0.20	0.37	0.19	0.31	0.43	0.24	0.33	P3
	0.23	0.41	0.24	1.00	0.23	0.45	0.21	0.38	0.21	0.34	0.22	0.41	0.22	0.42	0.21	0.34	0.45	0.23	0.36	
C4	0.29	0.19	0.41	0.23	1.00	0.26	0.42	0.24	0.35	0.22	0.33	0.19	0.39	0.21	0.34	0.20	0.24	0.43	0.27	C4
	0.32	0.21	0.44	0.23	1.00	0.24	0.43	0.24	0.37	0.22	0.37	0.21	0.43	0.22	0.35	0.21	0.24	0.43	0.24	
C3	0.20	0.31	0.23	0.42	0.26	1.00	0.23	0.44	0.21	0.36	0.19	0.33	0.21	0.39	0.20	0.34	0.39	0.29	0.41	C3
	0.22	0.34	0.23	0.45	0.24	1.00	0.23	0.45	0.22	0.39	0.22	0.38	0.22	0.43	0.21	0.37	0.43	0.25	0.42	
F4	0.24	0.18	0.31	0.20	0.42	0.23	1.00	0.25	0.41	0.24	0.27	0.18	0.35	0.20	0.35	0.21	0.20	0.37	0.28	F4
	0.28	0.20	0.35	0.21	0.43	0.23	1.00	0.24	0.42	0.24	0.32	0.21	0.39	0.22	0.37	0.22	0.22	0.39	0.25	
F3	0.20	0.26	0.21	0.34	0.24	0.44	0.25	1.00	0.24	0.43	0.19	0.29	0.21	0.36	0.21	0.36	0.32	0.26	0.44	F3
	0.23	0.31	0.22	0.38	0.24	0.45	0.24	1.00	0.24	0.44	0.22	0.34	0.22	0.41	0.22	0.39	0.38	0.24	0.45	
Fp2	0.23	0.19	0.27	0.19	0.35	0.21	0.41	0.24	1.00	0.25	0.24	0.18	0.32	0.19	0.36	0.21	0.19	0.33	0.26	Fp2
	0.26	0.21	0.30	0.21	0.37	0.22	0.42	0.24	1.00	0.24	0.29	0.21	0.36	0.21	0.38	0.22	0.21	0.34	0.24	
Fp1	0.20	0.24	0.20	0.29	0.22	0.36	0.24	0.43	0.25	1.00	0.18	0.26	0.20	0.32	0.21	0.36	0.28	0.22	0.41	Fp1
	0.23	0.29	0.22	0.34	0.22	0.39	0.24	0.44	0.24	1.00	0.22	0.32	0.22	0.37	0.22	0.40	0.34	0.22	0.42	
T6	0.35	0.19	0.38	0.20	0.33	0.19	0.27	0.19	0.24	0.18	1.00	0.19	0.36	0.19	0.30	0.18	0.22	0.29	0.19	T6
	0.37	0.21	0.40	0.22	0.37	0.22	0.32	0.22	0.29	0.22	1.00	0.22	0.40	0.21	0.33	0.22	0.22	0.31	0.21	
T5	0.19	0.36	0.20	0.38	0.19	0.33	0.18	0.29	0.18	0.26	0.19	1.00	0.18	0.38	0.18	0.31	0.32	0.21	0.27	T5
	0.22	0.39	0.22	0.41	0.21	0.38	0.21	0.34	0.21	0.32	0.22	1.00	0.21	0.41	0.21	0.35	0.37	0.21	0.31	
T4	0.28	0.19	0.37	0.20	0.39	0.21	0.35	0.21	0.32	0.20	0.36	0.18	1.00	0.19	0.38	0.19	0.21	0.32	0.22	T4
	0.31	0.21	0.40	0.22	0.43	0.22	0.39	0.22	0.36	0.22	0.40	0.21	1.00	0.21	0.40	0.21	0.22	0.35	0.22	
T3	0.19	0.30	0.20	0.37	0.21	0.39	0.20	0.36	0.19	0.32	0.19	0.38	0.19	1.00	0.19	0.39	0.32	0.22	0.32	T3
	0.22	0.34	0.22	0.42	0.22	0.43	0.22	0.41	0.21	0.37	0.21	0.41	0.21	1.00	0.21	0.41	0.37	0.22	0.37	
F8	0.24	0.18	0.29	0.19	0.34	0.20	0.35	0.21	0.36	0.21	0.30	0.18	0.38	0.19	1.00	0.19	0.19	0.29	0.22	F8
	0.26	0.21	0.31	0.21	0.35	0.21	0.37	0.22	0.38	0.22	0.33	0.21	0.40	0.21	1.00	0.21	0.21	0.30	0.22	
F7	0.20	0.26	0.19	0.31	0.20	0.34	0.21	0.36	0.21	0.36	0.18	0.31	0.19	0.39	0.19	1.00	0.27	0.21	0.32	F7
	0.23	0.30	0.22	0.34	0.21	0.37	0.22	0.39	0.22	0.40	0.22	0.35	0.21	0.41	0.21	1.00	0.32	0.21	0.35	
Pz	0.23	0.36	0.27	0.43	0.24	0.39	0.20	0.32	0.19	0.28	0.22	0.32	0.21	0.32	0.19	0.27	1.00	0.24	0.34	Pz
	0.23	0.39	0.25	0.45	0.24	0.43	0.22	0.38	0.21	0.34	0.22	0.37	0.22	0.37	0.21	0.32	1.00	0.24	0.38	
Cz	0.28	0.19	0.37	0.24	0.43	0.29	0.37	0.26	0.33	0.22	0.29	0.21	0.32	0.22	0.29	0.21	0.24	1.00	0.28	Cz
	0.30	0.20	0.39	0.23	0.43	0.25	0.39	0.24	0.34	0.22	0.31	0.21	0.35	0.22	0.30	0.21	0.24	1.00	0.24	
Fz	0.19	0.26	0.21	0.33	0.27	0.41	0.28	0.44	0.26	0.41	0.19	0.27	0.22	0.32	0.22	0.32	0.34	0.28	1.00	Fz
	0.22	0.30	0.22	0.36	0.24	0.42	0.25	0.45	0.24	0.42	0.21	0.31	0.22	0.37	0.22	0.35	0.38	0.24	1.00	
	O1	O2	P4	P3	C4	C3	F4	F3	Fp1	Fp2	T6	T5	T4	T3	F8	F7	Pz	Cz	Fz	
